# Changes in Vertical Mobility of the First Tarsometatarsal Joint During Gait After Combined Hallux Valgus Surgery Incorporating Proximal First Metatarsal Osteotomy

**DOI:** 10.3390/jcm15176514

**Published:** 2026-08-23

**Authors:** Yasunari Ikuta, Tsubasa Tashiro, Noriaki Maeda, Satoshi Arima, Honoka Ishihara, Sakura Oda, Yuki Tamura, Saori Ishibashi, Satoru Sakurai, Dan Moriwaki, Tomoyuki Nakasa, Nobuo Adachi

**Affiliations:** 1 Department of Orthopaedic Surgery, Hiroshima University Hospital, Hiroshima 7348551, Japan; 2 Sports Medical Center, Hiroshima University Hospital, Hiroshima 7348551, Japan; 3 Department of Sports Rehabilitation, Graduate School of Biomedical and Health Sciences, Hiroshima University, Hiroshima 7348553, Japan; 4 Department of Musculoskeletal Reconstruction and Biomaterials, Graduate School of Biomedical and Health Sciences, Hiroshima University, Hiroshima 7348551, Japan

**Keywords:** first tarsometatarsal joint, gait analysis, hallux valgus, hypermobility, medial cuneiform, metatarsal osteotomy, ultrasonography

## Abstract

**Background/Objectives**: Although proximal first metatarsal osteotomy is a common treatment for hallux valgus (HV), its effect on dynamic first tarsometatarsal (TMT) joint mobility remains unclear. Previous assessments have largely relied on static radiographic parameters, which may not capture functional joint kinematics. The aim of this study was to evaluate changes in first TMT joint mobility during the stance phase of gait using a synchronized ultrasound and three-dimensional motion capture system after combined hallux valgus surgery incorporating proximal first metatarsal osteotomy. **Methods**: We prospectively studied 23 feet of 21 female patients with symptomatic HV undergoing combined hallux valgus surgery incorporating proximal closing-wedge osteotomy. We also evaluated 11 feet of 11 healthy female volunteers as controls. Per clinical indications, additional concomitant procedures were performed for 21 of the 23 feet. Vertical displacement of the first metatarsal and medial cuneiform as well as the first TMT joint gap was measured throughout the stance phase of gait using a synchronized ultrasound and motion capture system. Pre- and postoperative kinematics (>1 year follow-up) were compared with those in the control group using one-dimensional statistical parametric mapping (SPM). **Results**: Radiographically, the HV and intermetatarsal angles significantly improved, while sagittal plane parameters showed no significant changes. SPM analysis revealed no significant differences in the first TMT joint gap or first metatarsal displacement among groups. Preoperatively, compared with the controls, HV feet exhibited significantly greater plantar displacement of the medial cuneiform during late terminal stance. Postoperatively, this abnormal displacement was significantly decreased, with no significant difference between groups. **Conclusions**: Combined hallux valgus surgery incorporating proximal first metatarsal osteotomy was associated with reduced dynamic medial column hypermobility, specifically shifting vertical displacement of the medial cuneiform towards patterns observed in healthy controls during the terminal stance phase. These functional improvements occurred despite minimal changes in static sagittal radiographic alignment. The findings highlight the importance of dynamic assessment for evaluation of postoperative medial column biomechanics.

## 1. Introduction

Hallux valgus (HV) is a common forefoot deformity that causes pain, functional limitation, and difficulty with footwear [1]. It is associated with malalignment of the first ray and altered load transfer through the medial column during gait. Mobility of the first tarsometatarsal (TMT) joint has been proposed as a contributing factor in the development and progression of HV. Recent research has reported a correlation between first TMT joint mobility and HV severity [2].

First TMT joint or first ray mobility has been assessed using radiographic, clinical, and cadaveric approaches. However, its definition and measurement method vary across studies, including radiographic translations or angular changes, clinical manual assessments, and cadaver-based kinematic measurements [3,4,5,6]. Radiographic studies have reported increased first TMT joint mobility in patients with HV and described articular characteristics of the joint that may be associated with mobility [5,7]. Cadaveric and prospective clinical studies have also supported an association between HV and increased first ray mobility [3,4]. Furthermore, biomechanical studies have shown that medial column stabilization procedures affect first ray kinematics [6]. Clinical studies have also suggested that first ray mobility may change after HV correction using proximal first metatarsal osteotomy combined with distal soft tissue procedures [8].

However, most prior assessments have been conducted under static or quasi-static conditions, including radiography, physical examination, or cadaver testing [9]. These approaches do not quantify first TMT joint kinematics during gait, when external loading and muscle activation vary throughout the stance phase. Accordingly, the effect of combined hallux valgus surgery incorporating proximal first metatarsal osteotomy on dynamic first TMT joint mobility remains unclear. To address this limitation, we developed a synchronized ultrasound and three-dimensional (3D) motion capture system to evaluate first TMT joint mobility during gait [10,11]. This method enables assessment of the motion of the first metatarsal and medial cuneiform, as well as their relative motion during the stance phase.

We hypothesized that combined hallux valgus surgery incorporating proximal first metatarsal osteotomy would reduce first TMT joint hypermobility during gait. Accordingly, the aim of this study was to investigate changes in first TMT joint mobility during the stance phase of gait after combined hallux valgus surgery incorporating proximal first metatarsal osteotomy. The first TMT joint gap and vertical displacement of the first metatarsal and medial cuneiform were evaluated as co-primary outcomes. The pre- and postoperative findings were compared with those for healthy controls.

## 2. Materials and Methods

### 2.1. Participants

This prospective comparative study included patients with HV and healthy controls. The study was conducted in accordance with the Declaration of Helsinki and approved by the Ethical Committee for Epidemiology of Hiroshima University (approval number: E2020-2187). All participants provided written informed consent.

We enrolled consecutive female patients with symptomatic HV who underwent proximal closing-wedge osteotomy (PCWO) of the first metatarsal at our hospital between May 2021 and February 2024. The inclusion criterion was painful HV refractory to nonoperative treatment. Feet with neuromuscular disorders that could affect gait were excluded; no patients met this criterion.

The HV group comprised 21 female patients (23 feet; 13 left, 10 right) with a mean age of 65.6 ± 9.7 years (range, 33–82) and a mean body mass index (BMI) of 23.0 ± 3.8 kg/m^2^. Postoperative measurements were obtained at least 1 year after surgery, with a mean follow-up of 13.7 months. Three patients (three feet) had rheumatoid arthritis. Per clinical indications, concomitant procedures were performed for 21 of the 23 feet; these included metatarsal shortening osteotomy (second metatarsal, 12 feet; third metatarsal, two feet; fourth metatarsal, one foot), corrective osteotomy of the fifth metatarsal (eight feet), Akin osteotomy (four feet), and arthrodesis of the second and third TMT joints (one foot).

The control group comprised 11 healthy female volunteers (11 feet; five left and six right) without HV. The mean age and BMI were 59.4 ± 8.6 years (range, 46–76) and 18.6 ± 2.7 kg/m^2^, respectively. Controls had no history of foot or ankle surgery, inflammatory joint disease, or current foot pain.

There was no significant difference in age between groups (*p* = 0.077); however, BMI was significantly lower in the control group than in the HV group (*p* = 0.0007).

### 2.2. Surgical Procedure

All patients underwent PCWO of the first metatarsal combined with distal soft tissue correction for HV using a standardized technique [12] under ultrasound-guided peripheral nerve blocks. A pneumatic tourniquet was applied to the leg with the patient in the supine position. As part of the lateral release procedure, the adductor hallucis tendon was detached from its insertion on the proximal phalanx. The lateral metatarsal–sesamoid suspensory ligament and lateral joint capsule were also selectively released according to the degree of contracture. Subsequently, through a medial longitudinal incision over the first metatarsophalangeal joint, the medial eminence of the first metatarsal head was exposed and resected, with preservation of the articular surface.

PCWO was performed for the first metatarsal. The osteotomy was planned so that the distal first metatarsal became parallel to the second metatarsal on the dorsoplantar view after correction. The distal fragment was supinated to correct pronation of the first metatarsal, and the osteotomy was temporarily fixed with a 1.5-mm Kirschner wire. A compression staple (DynaNite, 15 × 12 mm; Arthrex, Inc., Naples, FL, USA) was inserted from the lateral side of the osteotomy for compression at the lateral cortex. After fluoroscopic confirmation of alignment, an additional 3.0-mm headless double-ended screw (Wright Medical Technology, Inc., Memphis, TN, USA) was placed on the medial side for supplementary fixation. Finally, medial capsular plication was performed to maintain the hallux in the corrected position.

Additional procedures were performed as indicated. For shortening osteotomies of the second to fourth metatarsals, a transverse osteotomy was created at the metatarsal base perpendicular to the shaft, and a bone segment was resected to restore a smooth metatarsal parabola. The osteotomy was reduced and fixed with a compression staple (DynaNite, 15 × 12 mm, 13 × 10 mm, or 11 × 10 mm). For bunionette deformity, an oblique osteotomy or corrective osteotomy of the fifth metatarsal shaft was performed and fixed with a 2.5-mm headless screw (Wright Medical Technology, Inc.) or a compression staple (DynaNite, 11 × 10 mm).

Postoperatively, weight-bearing was permitted 1 week after surgery using a customized forefoot offloading shoe with arch support and an adjustable heel block (Nakamura Brace Co., Ltd., Oda, Shimane, Japan). At 5 weeks, the orthosis was replaced with an insole with a metatarsal pad and arch support. At 3 months, patients discontinued the insole and were allowed to resume activities without restriction.

### 2.3. Radiographic Evaluation

Weight-bearing dorsoplantar and lateral foot radiographs were obtained before surgery and at follow-up for postoperative assessment. The HV angle (HVA) and intermetatarsal angle (IMA) between the longitudinal axes of the first and second metatarsals were measured on the weight-bearing dorsoplantar radiographs (Supplementary Figure S1). Postoperative recurrence of HV deformity was defined as HVA > 20° [13].

On lateral radiographs, sagittal alignment of the foot and first ray was evaluated using the lateral talo-first metatarsal angle (Meary angle), the calcaneal pitch angle (CPA), the height between the medial cuneiform and the fifth metatarsal (MC–M5H), first metatarsal lift, and the first metatarsal–medial cuneiform angle (MMCA). First metatarsal lift was defined as the perpendicular distance from the inferior borders of the base of the first metatarsal to a line connecting the proximal and distal inferior borders of the medial cuneiform. The MMCA was defined as the angle formed by lines connecting the superior and inferior margins of the articular surfaces of the first metatarsal and medial cuneiform (Supplementary Figure S1) [14]. All measurements were performed using a digital picture archiving system (ShadeQuest/ViewR-DG version 1.26; Fujifilm Medical Co., Ltd., Tokyo, Japan).

### 2.4. Ultrasound and Gait Analysis

Vertical mobility of the first TMT joint during gait was evaluated using a synchronized ultrasound and 3D motion capture system, as described previously [10,11]. This method has demonstrated good to excellent intrarater reliability [11]. A B-mode ultrasound device (Art Us EXT-1H, Telemed, Vilnius, Lithuania) with a linear array probe (5–11 MHz, 60-mm field of view; Echo Blaster, Telemed) was used. The probe was longitudinally positioned over the dorsal aspect of the first TMT joint with an ultrasound gel pad and secured with an elastic band to minimize probe motion.

The ultrasound system was synchronized with an optical motion capture system (VICON MX T20-S; Vicon Motion Systems, Oxford, UK) and force plates (OR-6, 1000 Hz; AMTI, Watertown, MA, USA) embedded in the walkway. Reflective markers were placed on anatomical landmarks of the lower limbs and feet using the Vicon Plug-in Gait marker set (Vicon Motion Systems, Oxford, UK). The motion capture system was used for synchronized gait recording, whereas vertical motion of the medial cuneiform and first metatarsal was directly determined from the synchronized ultrasound images. No multisegment foot model was applied because the ultrasound probe, gel pad, and elastic securing band occupied the dorsal aspect of the foot, making reliable placement and tracking of additional foot markers difficult. Participants walked barefoot along the walkway at a self-selected comfortable speed. Ultrasound images, marker trajectories, and ground reaction forces (GRF) were simultaneously recorded during the gait trials.

**Figure 1:**
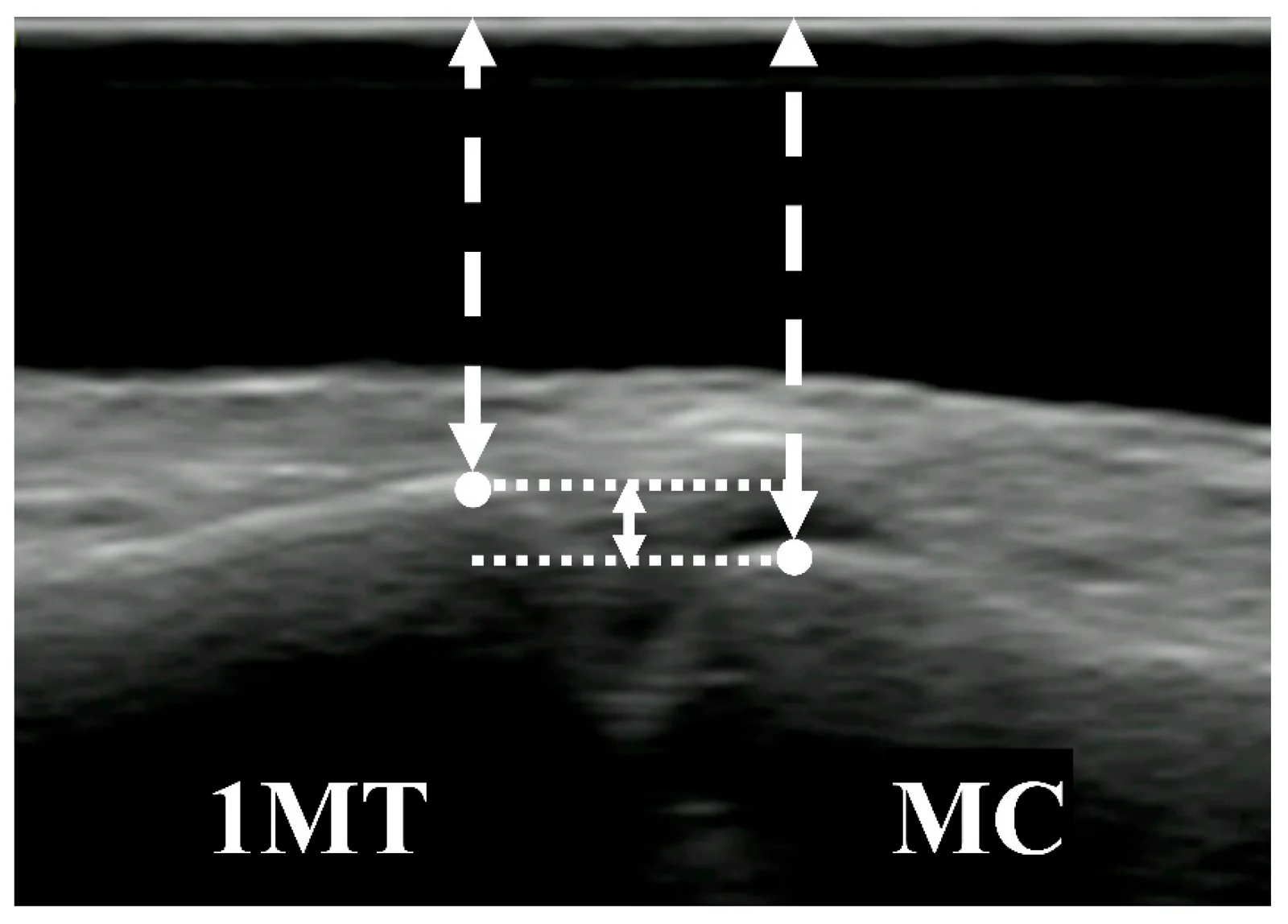
Representative ultrasonographic image of the first tarsometatarsal (TMT) joint. The dorsal cortices of the first metatarsal (1MT) and medial cuneiform (MC) are visualized. The vertical position of the 1MT and MC were defined as the perpendicular distance from the superior border of the image to the dorsal cortex of each bone (long dashed double-headed arrow). The first TMT joint gap was calculated as the difference between these vertical distances (solid double-headed arrow).

The stance phase was defined as the time from heel contact to toe-off using the vertical GRF data, and it was time-normalized to 100% of stance. Tracker 5.1.5 (Open Source Physics, https://www.compadre.org) was used to calculate the vertical translation of the first metatarsal and medial cuneiform from the ultrasonographic video. For each frame, the vertical positions of the first metatarsal and medial cuneiform were defined as the vertical distance from the superior border of the ultrasound image to the dorsal cortex of each bone. The vertical difference between these positions was defined as the first TMT joint gap (Figure 1). Vertical displacement of the first metatarsal and medial cuneiform and the first TMT joint gap was calculated across the stance phase. Three valid trials were collected for each participant, and mean values were used for analysis.

In the HV group, ultrasound and gait measurements were obtained preoperatively and at least 1 year postoperatively using the same protocol. In the control group, measurements were obtained once.

### 2.5. Statistical Analysis

For the control group, one foot per participant was randomly selected and included in the analysis to avoid within-subject dependence. Age and BMI were compared between the HV and control groups using Welch’s *t*-test. For the HV group, pre- and postoperative radiographic parameters were compared using a paired *t*-test. Walking speed was compared between the preoperative and postoperative conditions using a paired *t*-test, with significance set at *p* < 0.05. Each HV condition was then compared with the control group using Welch’s *t*-test, with significance set at *p* < 0.025 after Bonferroni correction for the two comparisons. These analyses were performed using IBM SPSS Statistics for Windows, Version 27.0 (IBM, Armonk, NY, USA).

For gait variables, time-series data across the entire stance phase were analyzed using one-dimensional statistical parametric mapping (SPM). SPM was selected because it enables evaluation of the normalized stance phase as a continuous trajectory rather than a series of discrete time points. First, to evaluate postoperative changes, pre- and postoperative curves within the HV group were compared using paired two-sample SPM *t*-tests, with significance set at *p* < 0.05. Second, to identify differences between the two groups, comparisons between the HV (pre- or postoperative) and control groups were performed using unpaired two-sample SPM *t*-tests. For these two control-related comparisons, Bonferroni correction was applied, and statistical significance was set at *p* < 0.025. In the SPM analysis, continuous intervals in which the SPM{t} trajectory exceeded the critical threshold determined using the random field theory were interpreted as suprathreshold clusters indicating significant differences between conditions. SPM analyses were performed using MATLAB (R2023a; MathWorks, Inc., Natick, MA, USA).

## 3. Results

**Figure 2:**
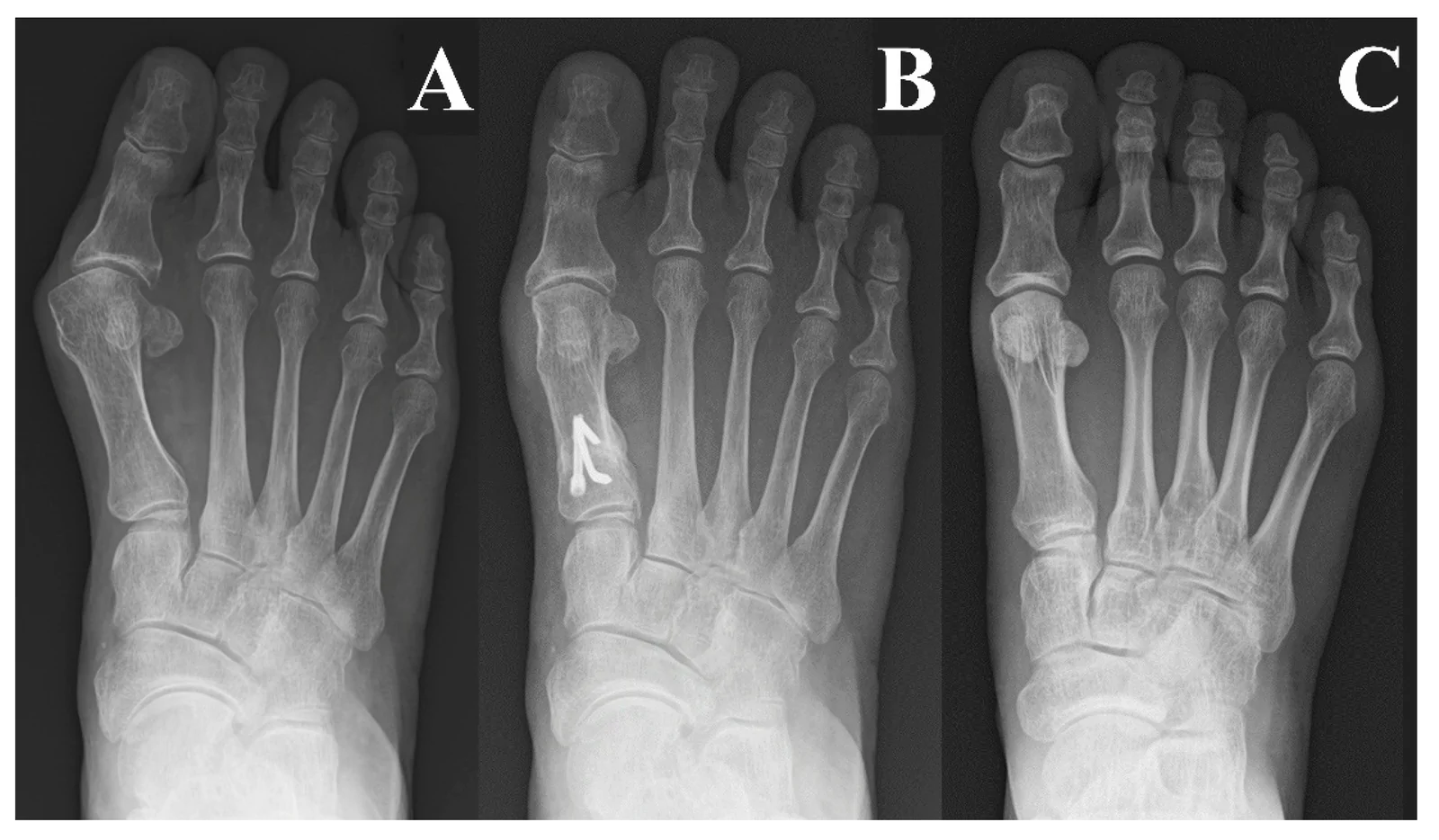
Representative weight-bearing dorsoplantar radiographs. (**A**) Preoperative radiograph of a case in the hallux valgus (HV) group. (**B**) Postoperative radiograph of a case in the HV group. (**C**) Radiograph of a case in the control group.

In the control group, the mean HVA and IMA were 10.7° ± 3.8° and 8.8° ± 1.7°, respectively. In the HV group, the mean HVA improved from 39.4° ± 8.7° preoperatively to 13.2° ± 5.9° at follow-up, while the mean IMA improved from 17.3° ± 3.3° to 8.0° ± 2.6°. Both HVA and IMA showed significant improvements after surgery (Figure 2). Recurrence of HV deformity, defined as HVA > 20°, was observed for three feet, all of which had severe deformity with preoperative HVA exceeding 50° (mean, 53.6°); the mean HVA for these feet was 24.6° at the final follow-up. On lateral radiographs, sagittal alignment parameters showed no significant changes between preoperative and follow-up assessments (Table 1).

Walking speed did not differ significantly between the preoperative and postoperative conditions, or between either HV condition and the control group (*p* = 0.52, 0.21, and 0.41, respectively).

**Figure 3:**
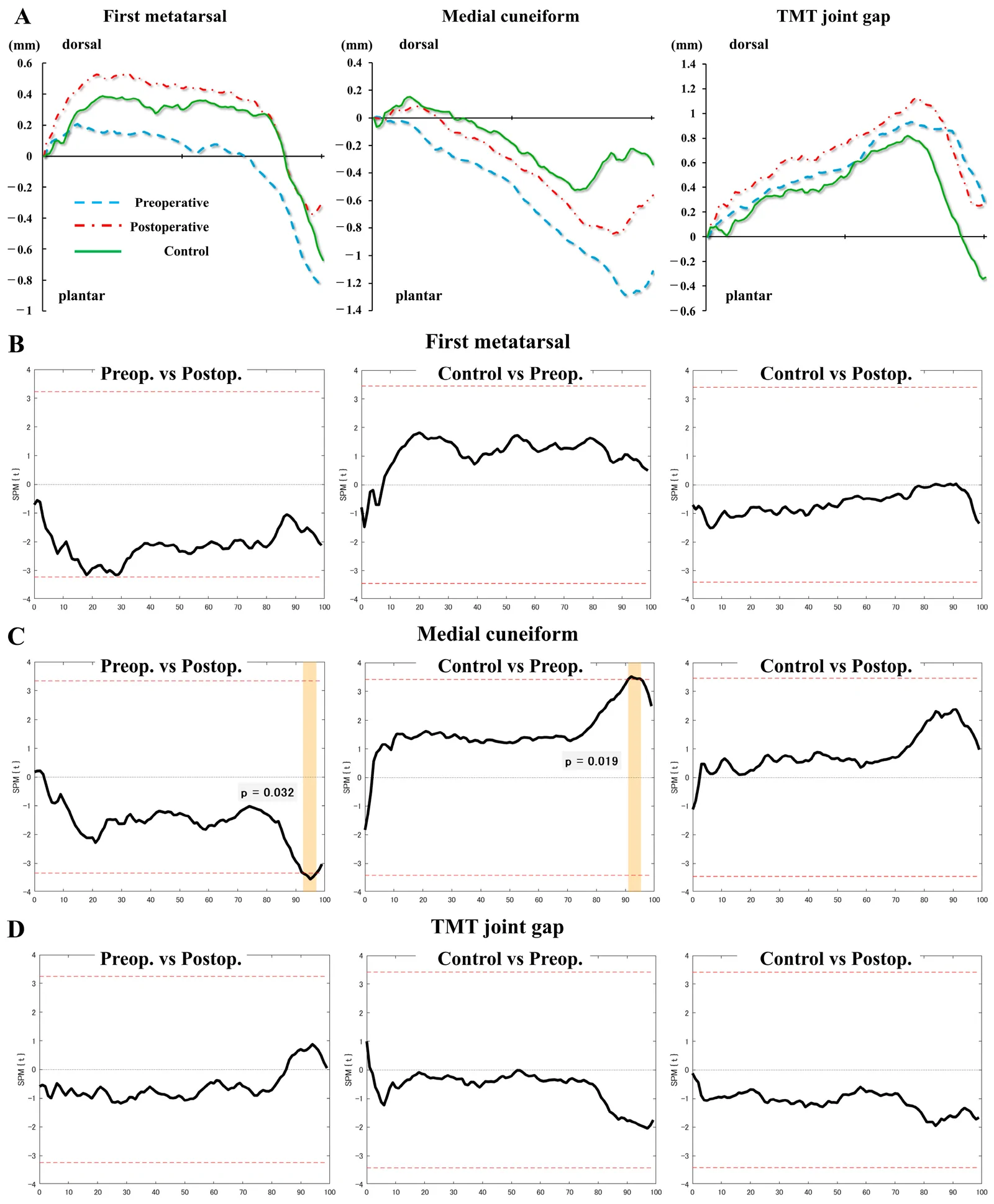
(**A**) Temporal changes in vertical displacement of the first metatarsal, medial cuneiform, and first tarsometatarsal (TMT) joint gap during the stance phase. The lines indicate the preoperative (blue long dashed), postoperative (red dash-dotted), and control (green solid) groups. The horizontal axis represents the stance phase, with the arrow indicating the direction of progression. (**B**–**D**) Results of statistical parametric mapping (SPM) analysis. The dashed line represents the critical threshold obtained from the SPM analysis. (**B**,**D**) With the numbers available, no significant difference in the first TMT joint gap or vertical displacement of the first metatarsal was observed. (**C**) For medial cuneiform displacement, orange shading indicates intervals with significant differences in the late terminal stance phase: preoperative vs. postoperative (*p* = 0.032) and preoperative vs. control (*p* = 0.019).

In the one-dimensional SPM analysis, with the numbers available, no significant difference was detected in the first TMT joint gap or vertical displacement of the first metatarsal among the preoperative, postoperative, and control conditions (Figure 3). In contrast, vertical displacement of the medial cuneiform showed significant suprathreshold clusters for the preoperative versus postoperative comparison from 92% to 97% of stance (*p* = 0.032) and the preoperative versus control comparison from 91% to 95% of stance (*p* = 0.019) (Figure 3). For the preoperative versus postoperative comparison, the mean difference within the suprathreshold window (92–97% of stance) was −0.60 mm (95% CI, −0.93 to −0.26), with a Cohen’s d_z_ of 0.72. For the preoperative versus control comparison, the mean difference within the suprathreshold window (91–95% of stance) was −0.75 mm (95% CI, −1.39 to −0.11), with a Cohen’s d of 0.83. With the numbers available, no significant difference in medial cuneiform displacement was observed between the postoperative and control conditions.

For the three feet with recurrence, qualitative inspection of individual medial cuneiform displacement curves demonstrated postoperative changes directionally consistent with those observed in the HV group. Given the small number of feet, no separate statistical analysis was performed.

## 4. Discussion

This study evaluated whether combined hallux valgus surgery incorporating proximal first metatarsal osteotomy for HV modified first TMT joint mobility during gait. The procedure achieved significant radiographic correction, with improvements in the HVA and IMA. However, radiographic sagittal alignment parameters showed no significant change. In time-series analysis, compared with the control group, the HV group demonstrated increased vertical displacement of the medial cuneiform in late terminal stance before surgery; this difference was not observed after surgery. These findings indicate that combined hallux valgus surgery incorporating proximal first metatarsal osteotomy is associated with a reduction in abnormal medial column motion during gait.

Radiographic studies support an association between medial column hypermobility and HV, although the causal pathway remains unclear [15,16]. Weight-bearing CT-based analyses have demonstrated differences in mobility of the first ray and adjacent midfoot joint between HV feet and controls [17,18,19]. Similarly, ultrasound-based assessments have suggested that greater first TMT joint mobility correlates with greater deformity severity [2]. Regarding postoperative changes, 3D analyses have reported reduced mobility of the first ray after proximal osteotomy [20], while another study reported early improvements in the windlass mechanism, indicating that surgical correction modifies function beyond static angular correction [21]. However, previous studies primarily quantified mobility under static or quasi-static loading. By directly evaluating gait, our study provides in vivo evidence of abnormality, specifically excessive plantar displacement of the medial cuneiform, particularly in late terminal stance. Postoperatively, these abnormalities decreased to levels comparable with those of healthy controls.

Our findings imply a discrepancy between static radiographic alignment and dynamic medial column mobility after osteotomy. Although dorsoplantar radiographs showed significant correction, sagittal plane radiographic parameters, including first metatarsal lift and the MMCA, did not show significant postoperative changes. In contrast, gait-based assessments demonstrated clear postoperative changes in medial cuneiform displacement. This aligns with reports indicating that static measures of instability do not necessarily correspond to dynamic kinematics during gait [22], and that radiographic parameters alone may be insufficient to characterize hypermobility of the first ray or its clinical relevance [23]. A recent study involving weight-bearing CT analyses further emphasized that medial column hypermobility may result from complex, multisegment motion rather than a single static alignment feature [24]. Thus, combined hallux valgus surgery incorporating proximal first metatarsal osteotomy induces meaningful dynamic changes that may not be readily detected by conventional static radiographic assessment. This highlights the utility of synchronized ultrasound and motion analyses for capturing postoperative functional biomechanics. Although the observed between-group difference in medial cuneiform displacement was small, it highlights the subtle nature of medial column hypermobility. Given the intrinsically rigid structure of the midfoot, even sub-millimeter dynamic displacements can significantly alter load transfer and joint congruity. Conventional static radiographs typically lack the sensitivity to detect such minute kinematic deviations; this may explain the difficulty in correlating radiographic hypermobility with clinical symptoms. By using high-resolution ultrasound synchronized with a motion capture system, we could detect subtle hypermobility that was previously undetected.

Beyond structural realignment, the observed improvements in medial column dynamics may be mediated by the functional restoration of extrinsic dynamic stabilizers. An altered line of action of the flexor hallucis longus (FHL) may contribute to phase-specific kinematics during terminal stance. The FHL dynamically stabilizes the medial longitudinal arch in late stance, generating plantarflexion and inversion moments that counteract GRF and stabilize the first ray [25]. FHL activity increases with walking speed and correlates with greater hallux loading during push-off [25,26]. In HV feet, lateral deviation of the FHL tendon creates a bowstringing effect [27]. FHL traction induces valgus alignment changes in the first ray, acting as a dynamic deforming force [28]. Consequently, proximal osteotomy may restore proper alignment for FHL function, contributing to the postoperative reduction in abnormal medial column motion. Furthermore, the peroneus longus modulates the mechanics of the first ray and counters deforming forces, particularly under axial loading [29,30]. Consistent with this, electrical stimulation of the peroneus longus acutely improves radiographic HV parameters; this supports its role in dynamic stabilization [31]. A recent study suggested that hypermobility arises from cumulative small movements across multiple bones rather than isolated motion of the first metatarsal [24]. These mechanisms remain hypothetical and warrant direct evaluation in future studies.

Medial column hypermobility in HV may not be confined to the first TMT joint alone, although it may involve joints surrounding the medial cuneiform. Abnormal motion has been reported at the intercuneiform and naviculocuneiform joints in HV [18,19]. Additionally, 3D-CT analyses indicate that corrective osteotomy can alter joint alignment beyond the first TMT joint, with measurable changes at the naviculocuneiform joint after HV correction [20]. Cadaveric kinematic data further support a multijoint construct of sagittal plane motion of the first ray; the naviculocuneiform, first TMT, and talonavicular joints reportedly contribute approximately 50%, 41%, and 9% of the total sagittal plane range of motion, respectively [6]. This is consistent with our finding that postoperative changes were most evident in medial cuneiform displacement. Osteotomy may therefore stabilize a medial cuneiform-centered joint complex rather than a single isolated joint. Because our measurements focused on the first TMT joint, future studies assessing adjacent joints are warranted to fully elucidate the effect of proximal osteotomy on medial column dynamics.

This study has some limitations. First, it was a single-center study with a relatively small sample size. In addition, only female patients were included. Thus, the findings cannot be generalized to male patients. Baseline BMI also differed between groups; this may have affected gait mechanics and limited the interpretation of between-group comparisons. In addition, differences in the thickness and deformation of the soft tissues between the ultrasound probe and underlying bone may have affected depth tracking, thereby introducing measurement artifacts. Although the use of a gel pad reduced direct probe compression, and the displacement variables were calculated as within-trial changes rather than absolute bone depths, the possibility of measurement errors arising from dynamic soft tissue deformation beneath the probe during gait cannot be completely excluded. Second, a small number of feet belonged to patients with rheumatoid arthritis. Inflammatory disease may affect joint laxity and gait mechanics, potentially confounding the observed kinematic patterns. Third, concomitant procedures were performed as clinically indicated for 21 of the 23 feet, and only two feet underwent surgery for HV without additional procedures. Therefore, the observed postoperative changes cannot be attributed to proximal first metatarsal osteotomy alone, and the findings should be interpreted as combined effects of the overall surgical reconstruction, including the proximal first metatarsal osteotomy and concomitant procedures. Finally, our analysis focused on vertical motion and relative displacement across the first TMT joint and did not capture the full 3-D kinematics of the medial column, including transverse plane and rotational components. Future studies should elucidate the complete 3D kinematics of the medial column, including rotational motion.

## 5. Conclusions

Combined HV surgery incorporating proximal first metatarsal osteotomy was associated with reduced medial cuneiform hypermobility during gait, particularly in late terminal stance. Although sagittal plane radiographic parameters did not change significantly, synchronized gait-based analysis revealed a postoperative reduction in medial cuneiform plantar displacement toward patterns observed in healthy controls. These findings suggest that proximal osteotomy modulates medial column dynamics during gait in ways that conventional static radiographs cannot capture.

## Figures and Tables

**Table 1:** Pre- and postoperative radiographic parameters.

	Preop. (*n* = 23)	Postop. (*n* = 23)	*p* Value
HVA (°)	39.4 ± 8.7 (35.6–43.2)	13.2 ± 5.9 (10.6–15.7)	<0.0001
IMA (°)	17.3 ± 3.3 (15.9–18.7)	8.0 ± 2.6 (6.9–9.1)	<0.0001
Meary angle (°)	8.5 ± 4.3 (6.7–10.4)	9.5 ± 4.0 (7.8–11.2)	0.26
CPA (°)	14.5 ± 4.0 (12.8–16.2)	13.7 ± 3.6 (12.2–15.3)	0.14
MC–M5H (mm)	5.8 ± 3.4 (4.3–7.2)	5.5 ± 2.9 (4.2–6.7)	0.66
First metatarsal lift (mm)	2.2 ± 1.2 (1.7–2.7)	2.1 ± 1.0 (1.7–2.6)	0.49
MMCA (°)	1.7 ± 0.9 (1.3–2.1)	1.4 ± 0.9 (1.0–1.8)	0.08

Data are presented as mean ± standard deviation (95% confidence intervals). HVA, hallux valgus angle; IMA, intermetatarsal angle; CPA, calcaneal pitch angle; MC–M5H, medial cuneiform–fifth metatarsal height; MMCA, first metatarsal–medial cuneiform angle. Significance was determined using a paired *t*-test.

## Data Availability

The de-identified data that support the findings of this study are available from the corresponding author upon reasonable request, subject to approval by the relevant institutional review board.

## Supplementary Materials

The following supporting information can be downloaded at: https://www.mdpi.com/article/10.3390/jcm15176514/s1, Supplementary Figure S1: Radiographic parameters. (A) Weightbearing dorsoplantar radiograph showing the hallux valgus angle (HVA) and intermetatarsal angle (IMA). (B) Weightbearing lateral radiograph showing the Meary angle and calcaneal pitch angle (CPA). (C) Medial cuneiform–fifth metatarsal height (MC–M5H), first metatarsal lift (Lift), and medial cuneiform–first metatarsal angle (MMCA).
