# Supplementary material for: Changes in Vertical Mobility of the First Tarsometatarsal Joint During Gait After Combined Hallux Valgus Surgery Incorporating Proximal First Metatarsal Osteotomy
Source: J Clin Med. 2026 Aug 23;15(17):6514. doi: 10.3390/jcm15176514 (PMC13565902; doi:10.3390/jcm15176514)
Supplement: Supplementary file 1 [file jcm-15-06514-s001.zip › jcm-4434065-supplementary.pdf]

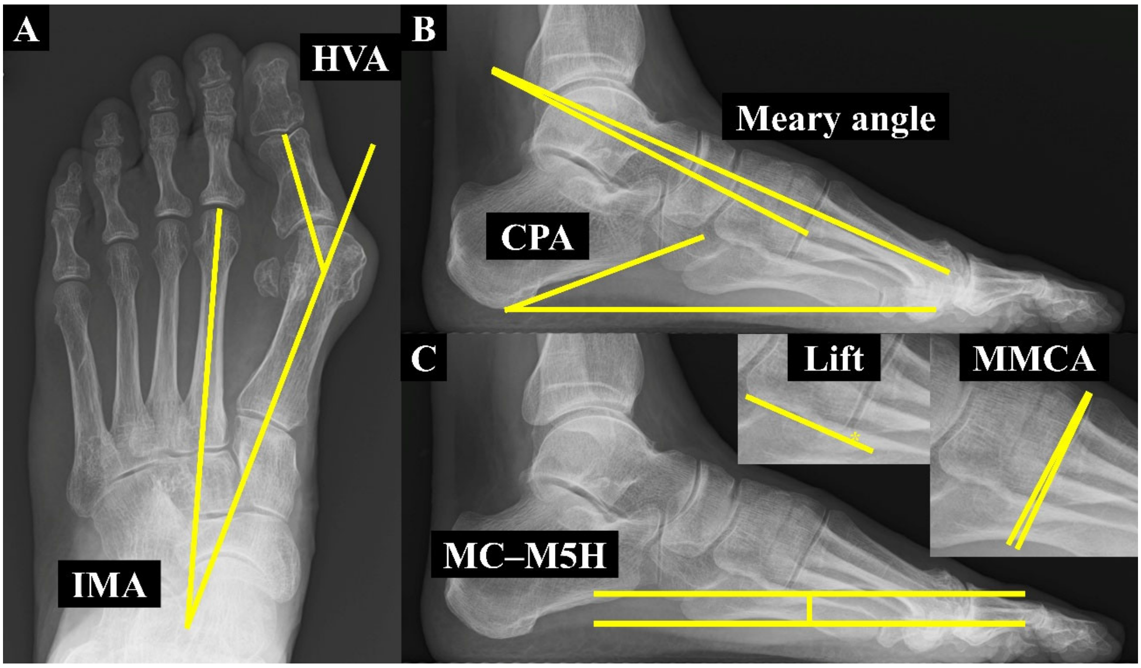

Supplementary Figure S1. Radiographic parameters. (A) Weightbearing dorsoplantar radiograph showing the hallux valgus angle (HVA) and intermetatarsal angle (IMA). (B) Weightbearing lateral radiograph showing the Meary angle and calcaneal pitch angle (CPA). (C) Medial cuneiform–fifth metatarsal height (MC–M5H), first metatarsal lift (Lift), and first metatarsal–medial cuneiform angle (MMCA).
